# Analysis of clinical characteristics and prognostic factors in 110 patients with nitrous oxide abuse

**DOI:** 10.1002/brb3.2533

**Published:** 2022-03-20

**Authors:** Miao Yu, Yue Qiao, Weishuai Li, Xiuying Fang, Han Gao, Dongming Zheng, Ying Ma

**Affiliations:** ^1^ Department of Neurology Shengjing Hospital of China Medical University Shenyang Liaoning Province China; ^2^ Department of Neurofunction Shengjing Hospital of China Medical University Shenyang Liaoning Province China

**Keywords:** clinical characteristics, nitrous oxide, outcomes, peripheral neuropathy, vitamin B12 deficiency

## Abstract

**Purpose:**

To review the clinical symptoms, auxiliary examination findings, and outcomes of patients with nitrous oxide (N_2_O) abuse, and analyze the factors that affect outcomes.

**Methods:**

Patients with N_2_O abuse treated in the Department of Neurology between January 2018 and December 2020 were included. The clinical data of these patients were collected, and follow‐up was conducted to determine the outcomes.

**Results:**

The average age of the 110 patients with N_2_O abuse was 21.4 ± 4.2 years (range: 14–33 years). Clinical presentation primarily included neurological symptoms, such as limb numbness and/or weakness (97%), psychiatric symptoms, changes in appetite, and skin hyperpigmentation. Laboratory test results were characterized by vitamin B_12_ deficiency (60%, 34 out of 57 cases) and high homocysteine level (69%, 31 out of 45 cases). Electromyography indicated mixed axonal and demyelination injury (92%, 80 out of 87 cases). Motor and sensory nerves were simultaneously involved, and injury primarily involved the lower limbs. One hundred and seven (97%) patients were clinically diagnosed with peripheral neuropathy, of whom 26 (24%) exhibited spinal abnormalities on magnetic resonance imaging, supporting a diagnosis of subacute combined degeneration. Treatment included N_2_O withdrawal and vitamin B_12_ supplementation. Reexamination of six patients indicated that treatment was effective. Follow‐up was completed for 51 patients. Thirty‐four patients (67%) recovered completely, 17 patients (33%) had residual limb numbness, and only one patient experienced relapse. Sex was an independent prognostic factor; the outcomes of female patients were better than that of male patients.

**Conclusion:**

The recreational use of N_2_O has largely expanded among youth in recent decades, which has become a growing public health concern in China. It highlights the importance of the recognition of various clinical symptoms, particularly limb numbness and/or weakness related to the cases of N_2_O abuse. The therapeutic administration of vitamin B_12_ supplementation and N_2_O withdrawal can make the overall prognosis good, especially for female patients.

## Graphical abstract

Follow‐up results of 51 patients in this study showed that patients with N_2_O abuse had an overall good prognosis, and their symptoms could be significantly improved in a short time. Sex was the N_2_O‐related independent factor among the three variables influencing neurological disease outcomes; female patients were more likely to achieve complete recovery than male patients (OR = 5.803, 95% CI: 1.289–26.125; *p *= 0.022).

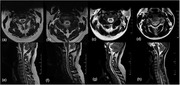

## 1 INTRODUCTION

Nitrous oxide (N_2_O) is a colorless gas that is often mixed with 30% oxygen for use as an anesthetic for dental and surgical operations, and its anesthetic effect is characterized by rapid onset and high safety. Ever since “laughing gas parties” became popular in the Victorian era, N_2_O has gradually become a popular inhaled drug because of its recreational use (Becker & Rosenberg, 2008; Brotman & Cullen, 1949; Weimann, 2003; Nagele et al., 2015; Van Amsterdam et al., 2015). In recent years, recreational N_2_O use has increased significantly both in China and abroad (Randhawa & Bodenham, 2016; The GDS Core Research, Team; Zheng et al., 2020). Currently, surveys on the prevalence and demographics of N_2_O abuse in China have not been comprehensive. The United States National Survey on Drug Use and Health estimates that 21% of teenagers who start using inhaled drugs start with N_2_O [OAS, 2009]. Short‐term use of high doses or long‐term use of N_2_O can lead to vitamin B_12_ deficiency, resulting in a series of neurological diseases, which primarily include peripheral neuropathy, myelopathy, and encephalopathy. Some patients may also experience psychiatric, emotional, and psychological changes, or even death (Blanco & Peters, 1983; Garakani et al., 2016; Keddie et al., 2018; Mancke et al., 2016; Patel et al., 2018; Pema et al., 1998; Sahenk et al., 1978; Van Amsterdam et al., 2015).

The use of N_2_O is not restricted in China. Although Internet search results for N_2_O are blocked in China, other options exist for finding suppliers online. N_2_O is becoming an increasingly important substance of abuse, although the public still lacks a more formal understanding of its adverse effects, which is also a major reason for its popularity. This study aimed to review the clinical characteristics and outcomes of patients with N_2_O abuse in order to improve the understanding of this condition by physicians and increase public awareness of the harm caused by N_2_O abuse.

## 2 PATIENTS AND METHODS

### 2.1 Research subjects and characteristics

Patients with N_2_O abuse treated in the Shengjing Hospital of China Medical University Department of Neurology (Clinic and Ward) between January 2018 and December 2020 were included in the study. This study complies with the Declaration of Helsinki and was approved by the Institutional Review Board of Shengjing Hospital (2020PS047K). The clinical data of patients were collected and included history of N_2_O exposure, clinical presentation, and results of laboratory, imaging, and electrophysiological examinations. Patients were followed up by telephone to determine outcomes. The content of the follow‐up included treatment plan, treatment duration, degree of recovery, whether relapse occurred, and changes in the results of laboratory, imaging, and electromyographic examinations. Patients were divided into a complete recovery group and an incomplete recovery group based on their status of recovery, and further analysis of factors that may affect outcomes was performed (Table 1).

**TABLE 1:** Variables and assigned values for eight factors with possible influence on outcomes

Influencing factor	Variable	Assigned values
Age (y)	*X*1	<18 = 0, ≥18 = 1
Sex	*X*2	M = 0, F = 1
Duration of abuse (months)	*X*3	<3 = 0, ≥3 = 1
Duration of medical history (months)	*X*4	<3 = 0, ≥3 = 1
Vitamin B_12_ deficiency	*X*6	No = 0, Yes = 1
Elevated homocysteine	*X*7	No = 0, Yes = 1
Anemia	*X*8	No = 0, Yes = 1
Spinal MRI	*X*9	Normal = 0, Abnormal = 1
Complete recovery	*Y*	No = 0, Yes = 1

MRI, magnetic resonance imaging.

### 2.2 Statistics

SPSS 26.0 software (IBM SPSS Statistics for Windows, Armonk, NY: IBM Corp.) was used for data processing and analysis. Measurement data conforming to the normal distribution are shown as mean ± standard deviation (*X* ± *S*), and the Student's *t*‐test was used for statistical analysis. Measurement data not conforming to the normal distribution are shown as median and interquartile range [*M* (P25, P75)], and the Mann–Whitney *U* test was used for statistical analysis. Count data are expressed as relative proportion (%) or rate (%), and the chi‐squared test was used for statistical analysis. For prognostic factors, univariate analysis was conducted first, the independent variables were initially screened based on *p* < 0.1, and then logistic regression analysis was conducted. Differences with *p* < 0.05 were considered statistically significant.

## 3 RESULTS

### 3.1 General demographic data and clinical presentation

Demographic data and clinical presentation of the patients are shown in Table 2. A total of 110 patients (57 males and 53 females) were included. Of the 110 patients, 96 patients came from the neurology clinic. Fourteen patients came from the neurology ward and they were admitted to the ward after going through the neurology clinic. The average patient age was 21.4 ± 4.2 y (range: 14–33 y). None of the patients had a personal or family history of neurological or psychiatric diseases. All patients had a history of N_2_O exposure; the average duration of exposure was 0.5–72 (12.5 ± 4.2) mo. The most common clinical presentations were limb numbness and/or weakness (97%), of which the symptoms involved all four limbs in 64% of patients. Other neurological symptoms included difficulty walking (12%), headache or dizziness (9%), involuntary movements (7%), constipation (5%), urinary retention (3%), urinary incontinence (2%), memory loss (3%), epilepsy (2%), and foot drop (2%). Some patients experienced psychiatric symptoms, such as mood disorders (2%), presenting as anxiety, irritability, or depressed mood; sleep disorders presenting as insomnia (3%) or hypersomnia (1%); and a few patients even developed hallucinations (3%) and behavioral abnormalities. The most common neurological signs were decreased muscle strength (83%), followed by superficial sensory disturbances (80%), decreased tendon reflex (71%), deep sensory disturbances (64%), and positive Romberg's sign (62%), with increased tendon reflex (9%) and positive Babinski sign (7%) in a small number of patients.

**TABLE 2:** Demographic data and clinical presentation

	*X* ± *S N*/Total *N* (%) (range)
Sex	57 male, 53 female
Age (y)	21.4 ± 4.2 (14–33)
Duration of N_2_O abuse (mo)	12.5 ± 4.2 (0.5–72)
Neurological symptoms	
Limb numbness or weakness	107 (97%)
All limbs	70 (64%)
Lower limbs	37 (34%)
Difficulty walking	13 (12%)
Headache or dizziness	9 (8%)
Involuntary movements	7 (6%)
Constipation	5 (5%)
Memory loss	3 (3%)
Urinary retention	3 (3%)
Epilepsy	2 (2%)
Foot drop	2 (2%)
Urinary incontinence	2 (2%)
Psychiatric symptoms	
Mood disorders	2 (2%)
Insomnia	3 (3%)
Hypersomnia	1 (1%)
Hallucinations	3 (3%)
Personality changes	1 (1%)
Other symptoms	
Chest tightness or pain	5 (5%)
Increased appetite	12 (11%)
Decreased appetite	9 (8%)
Skin hyperpigmentation	3 (3%)
Physical examination	
Decreased muscle strength	91 (83%)
Superficial sensory disturbances	88 (80%)
Deep sensory disturbances	70 (64%)
Decreased tendon reflex	78 (71%)
Increased tendon reflex	10 (9%)
Positive Romberg's sign	68 (62%)
Positive Babinski sign	8 (7%)

*Notes*: *N*/Total *N* (%), abnormal number/total number (%).

### 3.2 Laboratory, imaging, and electromyographic examinations

**FIGURE 1:**
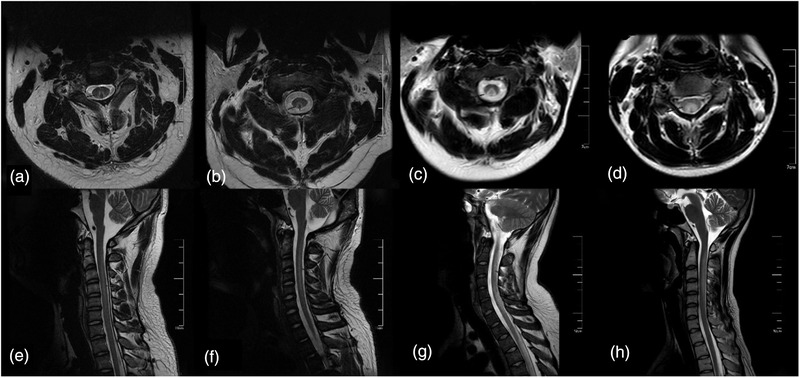
Spinal magnetic resonance imaging. (a–d) Transverse sections of spinal cord showing “V,” inverted “V,” triangle, and elliptical signals. (e–h) Sagittal sections showing high T2 signal lesion involving the dorsal longitudinal segment of the spinal cord

The results of laboratory, imaging, and electromyographic examinations are shown in Table 3. Vitamin B_12_ level was tested in 66 patients (333.6 ± 396.7 pg/ml). Among them, nine patients had self‐administered vitamins before seeking medical help, and their vitamin B_12_ levels were very high (1152 ± 527.8 pg/ml). In reality, 60% (34 out of 57 cases) of patients were deficient in vitamin B_12_. Of the 45 patients tested for high homocysteine level, 69% (31 out of 45 cases) exhibited high homocysteine levels (44.6 ± 42.1 μmol/L). Of the 71 patients tested for hemoglobin (133.3 ± 20.7 g/L), 35% (25 out of 71 cases) exhibited anemia. In these patients, folic acid levels were within the normal range, and 20% (five out of 25 cases) of these patients presented with hypochromic macrocytic anemia, with a mean corpuscular volume of 107.1 ± 5.2 fl (normal value 83–101 fl). Tests for thyroid function, rheumatoid arthritis‐associated antibodies, antineutrophil cytoplasmic antibodies, tumor biomarker, and human immunodeficiency virus (HIV) were performed in some patients, and all results were normal or nonspecific. Spinal magnetic resonance imaging (MRI) revealed a long T2 signal on the dorsal side of the spinal cord in 52% (26 out of 50 cases) of patients. The signal was generally located in the neck, in a “V,” inverted “V” triangle, or elliptical shape (Figure 1). Head MRI revealed abnormalities in 55% (six out of 11 cases) of patients, of whom three exhibited brain atrophy, and the other three exhibited demyelination, primarily distributed in the frontal lobe. A total of 87 patients underwent electromyography (EMG) examination of four sensory nerves and four motor nerves. Prolonged distal latency and decreased amplitude indicate axonal injury, and decreased nerve conduction velocity indicates demyelination. The results of the examination showed that peripheral neuropathy associated with N_2_O abuse were primarily mixed axonal and demyelination injury, accounting for 92%, followed by demyelination, accounting for 8%. Motor and sensory nerves were often simultaneously involved, and lower limb involvement was significant (*p *< 0.0001).

**TABLE 3:** Laboratory, imaging, and electromyography results

	*X* ± *S*	*N*/Total *N* (%)	Range
Vitamin B12 (180–914 pg/ml)	333.6 ± 396.7	34/57 (60%)	6.0–1500
HCY (0–15 μmol/L)	44.6 ± 42.1	31/45 (69%)	2.6–158.4
Hb (130–172 g/L)	133.3 ± 20.7	25/71 (35%)	70–168
Abnormal spinal cord MRI		26/50 (52%)	
Abnormal brain MRI		6/11 (55%)	
EMG			
Demyelinating		7/87 (8%)	
Mixed		80/87 (92%)	
Abnormal motor nerve		178/308 (58%)	
Abnormal sensory nerve		199/326 (61%)	
Abnormal upper limb nerve		106/311 (34%)	
Abnormal lower limb nerve		271/323 (84%)	

*Notes*: *N*/Total *N* (%), abnormal number/total number (%); EMG, electromyography; Hb, hemoglobin; HCY, homocysteine; MRI, magnetic resonance imaging; Mixed, both axonal damage and demyelinating.

**TABLE 4:** Univariate analysis of nitrous oxide (N_2_O)‐related factors influencing neurological disease outcomes

Influencing factor	Complete recovery group (*n* = 34)	Noncomplete recovery group (*n* = 17)	*c* ^2^	*p*
Age (y)			7.013	0.008
<18	11	0		
≥18	23	17		
Sex			3.279	0.070
M	17	13		
F	17	4		
Duration of N_2_O abuse (months)			1.29	0.256
<3	7	6		
≥3	27	11		
Duration of medical history (months)			3.632	0.057
<3	31	12		
≥3	3	5		
Vitamin B_12_ deficiency			0.039	0.842
No	15	7		
Yes	19	10		
Elevated homocysteine			0.842	0.306
No	11	8		
Yes	23	9		
Anemia			0.177	0.674
No	22	12		
Yes	12	5		
Spinal MRI			1.457	0.227
Normal	18	12		
Abnormal	16	5		

MRI, magnetic resonance imaging.

**TABLE 5:** Logistic regression analysis of N_2_O‐related factors influencing neurological disease outcomes

Influencing factor	*B*	SE	Wald	*p*	OR	95%CI
Sex	1.758	0.768	5.246	0.022	5.803	1.289–26.125
Age	20.743	11,506.4	0	0.999	1019884948	0
Duration of medical history	1.706	1.01	2.851	0.091	5.508	0.760–39.917

CI, confidence interval; OR, odds ratio; SE, standard error.

Based on *p* < 0 .1, the three independent variables of age, sex, and duration of medical history were selected for univariate analysis (Table 1 and Table 4). Further logistic regression analysis (Table 5) showed that sex was the N_2_O‐related independent factor among the three variables influencing neurological disease outcomes; female patients were more likely to achieve complete recovery than male patients (OR = 5.803, 95% CI: 1.289–26.125; *p *= 0.022).

### 3.3 Diagnosis and treatment

Based on clinical presentation and signs, 107 patients (97%) were clinically diagnosed with peripheral neuropathy. Eighty‐seven patients underwent EMG in order to obtain further electrophysiological evidence for peripheral neuropathy. Spinal MRI of 26 of these patients revealed posterior spinal cord injury, supporting subacute combined degeneration. All patients were treated with N_2_O withdrawal and intramuscular injection or oral supplementation of vitamin B_12_ and vitamin B_1_; some patients were administered folic acid, vitamin B_6_, and other nerve supplements.

### 3.4 Follow‐up

**FIGURE 2:**
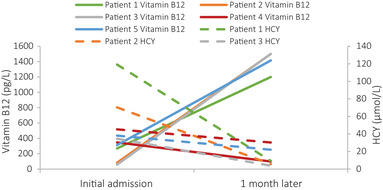
Changes in vitamin B_12_ and homocysteine (HCY) levels in patients with nitrous oxide abuse

**FIGURE 3:**
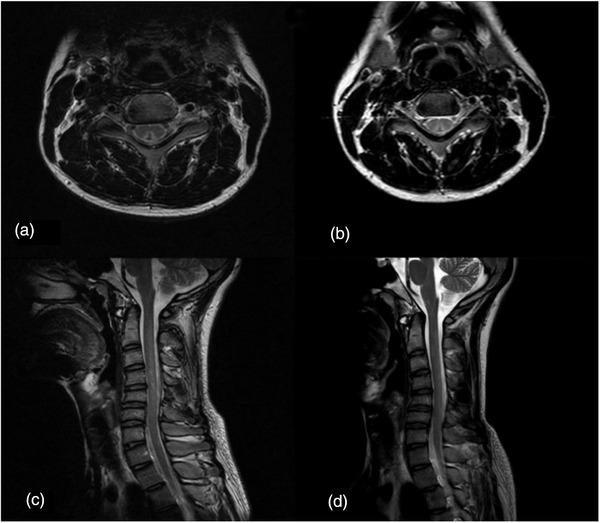
(a, c) Magnetic resonance image (MRI) examination of patients with nitrous oxide abuse at initial consultation showed increased T2 signal in the spinal cord at the C3–C6 level. (b, d) MRI reexamination after 1 month showed reduction of increased T2 signal in the spinal cord at the C3–C6 level

**FIGURE 4:**
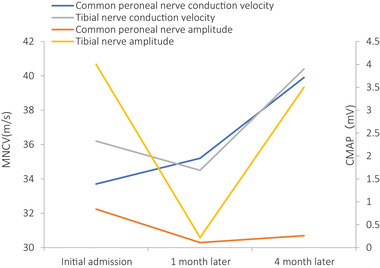
Changes in compound muscle action potential (CMAP) and motor nerve conduction velocity (MNCV) in patients with nitrous oxide abuse

During the treatment period, six patients underwent re‐examination: at 1 mo after treatment, four patients underwent blood tests only, one underwent blood tests and cervical spine MRI, and one underwent EMG only; this patient also underwent EMG re‐examination at 4 mo after treatment. Vitamin B_12_ tests indicated that four patients reached the normal reference range or above, and homocysteine tests indicated that five patients achieved a significant decrease from elevated levels (Figure 2). Cervical spine MRI reexamination indicated that the abnormal signal of the spinal cord was reduced from before (Figure 3). One patient who underwent EMG reexamination underwent the initial EMG examination after limb weakness at 10 d; the result peripheral nerve damage, sensory motor fiber involvement, myelin sheath and axon involvement, and the lower limbs as the principal site affected. Symptoms were not significantly improved after 1 month of treatment, and EMG reexamination indicated that the amplitude and nerve conduction velocity of the motor nerves were still significantly decreased from before. Symptoms improved significantly after 4 months of treatment, and EMG reexamination indicated that nerve conduction velocity and amplitude of the motor nerves had improved to varying degrees (Figure 4). All patients underwent follow‐up by telephone after data collection. The most common reason for loss to follow‐up was the inability to reach the patient by telephone. Eventually, telephone follow‐up was completed in only 51 patients (46%). Thirty‐four of these patients (67%) recovered; their average age was 21.0 ± 4.2 y, their duration of medical history was 43.6 ± 69.8 d (range: 3–365 d), and their treatment duration was 77 ± 74.1 d (range: 15–365 d). Seventeen of the patients (33%) experienced sequelae, which primarily included limb numbness; their average age was 21.7 ± 2.8 y, their duration of medical history was 72.2 ± 113.9 d (range: 3–365 d), and their treatment duration was 83.1 ± 60.2 d (range: 30–180 d). Only one patient relapsed at 6 mo after complete recovery of symptoms. The duration of relapse was 1 y, and the frequency of abuse was about 1–2 times/year; the amount consumed was small each time, other details are unknown, and no obvious clinical symptoms have appeared.

## 4 DISCUSSION

N_2_O has euphoric effects in addition to anesthetic effects, and has been used as a substitute for benzodiazepines to relieve withdrawal syndromes caused by cocaine, alcohol, and other substances. It has also been recommended for the treatment of refractory depression [Daynes & Gillman, 1994; Gillman & Lichtigfeld, 1985; Gillman et al., 2006; Gillman et al., 2007; Nagele et al., 2015]. The mechanism by which N_2_O produces euphoria remains poorly understood and may be due to the inhibition of N‐methyl‐d‐aspartate (NMDA) receptors, similar to the noncompetitive NMDA receptor antagonist ketamine (Jevtović‐Todorović et al., 1998; Maze & Fujinaga, 2000; Garakani et al., 2016; Richardson & Shelton, 2015; Weimann, 2003). In recent years, the recreational use of N_2_O has increased significantly, which may be because N_2_O is a legal drug in most countries and is widely available at a low price. A survey in the United States found that N_2_O was the first drug of abuse in 21% of young people. The majority of those who abuse N_2_O are young people, and the most common way of consumption is through balloon inhalation (OAS, 2009; Drug Misuse: Findings from the, 2016/17 Crime survey for England and Wales; Nabben et al., 2014; Kaar et al., 2016). The average age of the 110 patients in this study was 21.4 ± 4.2 years (range: 14–33 years), consistent with the results of previous research.

The earliest reports on N_2_O abuse in the literature can be traced back to 1961 [Enticknap, 1961]. In 1978, Layzer et al. were the first to report nerve injury caused by N_2_O exposure. Three cases of peripheral neuropathy caused by N_2_O were diagnosed through nerve conduction testing. Previous studies showed that peripheral neuropathy and myelopathy are among the most common N_2_O‐related neurological disorders (Hathout & El‐Saden, 2011; Hsu et al., 2012; Layzer et al., 1978; Hew et al., 2018; Fang et al., 2019; ). Short‐term use of high doses of N_2_O and long‐term use of N_2_O are more likely to cause neurological symptoms. The severity of clinical symptoms is associated primarily with the amount of N_2_O inhaled (Cheng et al., 2013; Vasconcelos et al., 2006). The present study showed that the most common neurological symptoms were limb numbness and weakness (97%) and decreased muscle strength (83%), which appeared primarily in the lower limbs. Therefore, when young patients with limb numbness and weakness are encountered in the clinic, it is important that N_2_O abuse be considered and medical history ascertained. In addition, N_2_O abuse should be differentiated from other causes of neuropathy, such as diabetic peripheral neuropathy and alcoholic peripheral neuropathy. In addition to neurological symptoms, some patients also present with psychiatric symptoms, such as visual hallucinations, auditory hallucinations, delusions, disordered speech, and violence toward persons or objects. The possibility of N_2_O abuse should not be overlooked when young patients with acute psychiatric symptoms are encountered in the clinic. A small number of patients present with urinary incontinence or urinary retention, which may be associated with autonomic nerve involvement. Notably, we found changes in appetite in 21 patients (19%), some of whom presented with excessive appetite accompanied by significant weight gain, and others with decreased appetite. The specific mechanism by which N_2_O affects appetite remains to be studied further. A small number of patients presented with skin hyperpigmentation, primarily on the fingers, toes, and back. Past case reports have indicated that skin hyperpigmentation may be the only first symptom in patients with N_2_O abuse, and its appearance may be due to vitamin B_12_ deficiency leading to decreased intracellular reduced glutathione levels. Reduced glutathione inhibits tyrosinase, and decreased reduced glutathione stimulates epidermal melanocytes to produce melanin, resulting in skin hyperpigmentation (Chiang et al., 2013; Gilliam & Cox, 1973). The clinical manifestations of patients with N_2_O abuse vary and may involve neurological, psychiatric, autonomic nervous system, and even dermatological symptoms. For clinicians, the ability to identify the possible clinical manifestations of N_2_O abuse will prevent delays in diagnosis and treatment.

N_2_O toxicity is believed to be associated with its interaction with vitamin B_12_. Long‐term exposure to N_2_O can cause vitamin B_12_ deficiency, which is closely associated with neurological, psychiatric, dermatologic, and hematologic symptoms (Banks et al., 1968; Chanarin, 1980; Oh & Brown, 2003; Pema et al., 1998). Vitamin B_12_ plays a key role in methylation reactions and DNA synthesis. Methionine synthesis requires methylcobalamin, a reduced form of vitamin B_12_, for the conversion of homocysteine into methionine. This step preserves donor S‐adenosylmethionine for later methylation and connects to the DNA synthesis pathway through the production of tetrahydrofolic acid. Once N_2_O is decomposed into free nitrogen and oxygen, it rapidly oxidizes vitamin B_12_ and irreversibly inactivates it, inhibiting methionine synthase activity and blocking methylation and DNA synthesis, thus causing central and peripheral nerve demyelination (Hathout & El‐Saden, 2011; Kräutler, 2005; Pema et al, 1998; Reynolds, 2006). In addition to affecting vitamin B_12_, N_2_O may also exert its neurotoxic effects through an antagonism of NMDA receptors (Maze M, Fujinaga, 2000; Garakani et al., 2016; Keddie et al., 2018). Moreover, homocysteine accumulation, oxidative stress damage, and changes in cytokine and growth factor levels are also believed to be associated with N_2_O‐induced neuropathy (Veber et al., 2006; Savage & Ma, 2014; Singh et al., 2015). The causes of the psychiatric symptoms remain poorly understood. Hutto et al. believed that increased synthesis of tetrahydrobiopterin (BH_4_) affected the rate of monoamine (dopamine, norepinephrine, and serotonin) synthesis, thereby inducing psychiatric symptoms, whereas others believed psychiatric symptoms to be associated with cerebral hypoxia, methemoglobinemia, low arterial partial pressure of oxygen, and acidosis (Brodsky & Zuniga, 1975; Hutto, 1997; Scalabrino, 2005).

As mentioned above, N_2_O irreversibly induces the oxidation of vitamin B12, which not only blocks methylation and DNA synthesis, but also leads to homocysteine accumulation. The findings of the present study also suggest that elevated homocysteine level is very common in patients with N_2_O abuse, as 69% of the patients exhibited hyperhomocysteinemia. Decreased vitamin B_12_ was also a common abnormal test result, but it was not as high as the rate of abnormal level of homocysteine, consistent with the findings of previous studies (Hannibal et al., 2016; Li et al., 2016; Oussalah et al., 2019]. This may be because many patients have learned about the possible adverse consequences of N_2_O inhalation and its prevention through their social circles and take vitamin B_12_ supplementation on their own before initial consultation. Some patients with normal serum vitamin B_12_ levels have functional vitamin B_12_ deficiency and clinical symptoms. Serum vitamin B_12_ levels do not represent intracellular levels of vitamin B_12_ nor do it represent the existence of functional vitamin B_12_ deficiency. Cyclocobalamine is the gold standard test to evaluate vitamin B_12_ deficiency, but it is impossible, because it is a retrospective study and was limited by conventional test methods. Increased homocysteine or methylmalonic acid levels indicates a lack of functional vitamin B_12_ at the cellular level (Chiang et al., 2013). Therefore, homocysteine is more suitable as a test indicator than vitamin B_12_ in patients who are presumed to have N_2_O abuse. Imaging examination of patients with N_2_O abuse is characterized by a long T2 signal on the dorsal side of the spinal cord that is located primarily in the neck and presents as a symmetrical high‐intensity signal in the shape of an inverted “V,” triangle, or ellipse. The cervical spinal cord is the most common site of involvement on MRI. The most common vertebral levels involved are C3 and C4, extending to the thoracic spine in severe cases. The cervical spinal cord is most susceptible to involvement, possibly because the density of myelinated fibers in the fasciculus gracilis is highest in the cervical spinal cord (Ohnishi et al., 1976). Subacute combined degeneration caused by vitamin B_12_ deficiency is characterized by the involvement of the posterior and lateral spinal cord. However, we found that subacute combined degeneration associated with N_2_O abuse primarily affected the posterior spinal cord and rarely involved the lateral spinal cord. Impairment of the fasciculi graciles and fasciculi cuneati affects deep sensory conduction. A total of 70 (64%) patients exhibited deep sensory disturbances, which primarily presented as ataxia, difficulty walking, and positional and vibrational disturbances. Abnormal brain MRI was uncommon; three patients presented with brain atrophy, but only one patient presented with mild memory loss, and the other three patients exhibited demyelination, primarily in the frontal lobe. This may be explained by impaired methylation of myelin phospholipids caused by N_2_O abuse leading to demyelination of nerves in the central nervous system [Pema et al., 1998; Maze & Fujinaga, 2000]. In this study, only 11 patients underwent MRI examination of brain, but the abnormal rate was relatively high (55%), which may be related to the symptoms of brain injury in these patients before the examination. However, we should recognize that it is necessary to perform brain/spinal MRI to help determine whether there is brain/spinal cord injury, and the severity and prognosis of diseases.

EMG is of great significance for the diagnosis of peripheral neuropathy. In the present study, EMG of 87 patients with N_2_O abuse revealed abnormalities of varying degrees, characterized by mixed axonal and demyelination injury. Motor and sensory nerves were often simultaneously involved, and there was significant injury in the lower limbs. These results are consistent with those of previous reports of N_2_O‐related peripheral neuropathy and the characteristics of most electromyographic changes in peripheral neuropathy (Vasconcelos et al., 2006; Li et al., 2016; Zheng et al., 2020). EMG examination is beneficial for the diagnosis of N_2_O‐related peripheral neuropathy, determining the degree of injury, and whether the injury is in the acute or subacute phase. Thus, clinicians should consider the history of N_2_O exposure together with the results of laboratory and imaging examinations in diagnosing, taking care to differentiate it from other acute motor axonal neuropathies, diabetic peripheral neuropathy, alcoholic peripheral neuropathy, and polyneuropathy following HIV infection.

The primary treatment for N_2_O abuse is its withdrawal and vitamin B_12_ supplementation by intramuscular injection or oral administration. Laboratory, imaging, and EMG examinations during treatment indicated that the treatment was effective, and the EMG results were consistent with clinical symptoms. After 1 mo of treatment, the symptoms were not significantly improved, and EMG reexamination indicated that the amplitude and conduction velocity of the motor nerves were still significantly decreased from before. After 4 mo of treatment, the symptoms were significantly improved, and EMG reexamination indicated that the conduction velocity and amplitude of the motor nerve had improved to varying degrees. The follow‐up results of 51 patients in the present study indicated that the patients generally had good outcomes and that their symptoms were significantly improved within a short period of time. Only 17 patients (33%) had residual limb numbness, but their daily living activities were largely unaffected. Sex was an independent prognostic factor, but the specific reasons remain to be studied further. There was only one case of relapse among the patients who underwent follow‐up, which may indicate that N_2_O withdrawal is relatively easy. The follow‐up rate of the present study was only 46%, which may be insufficient to reflect the true outcomes and relapse rate of patients with N_2_O abuse.

As this study was retrospective, it had the following limitations: the clinical data is incomplete; the quantity of N_2_O intake is uncertain; lack of cyclocobalamin testing; low rate of MRI examination; low reexamination rate; low telephone follow‐up rate. These are the limitations of this study, which need to be further improved in future prospective research.

## 5 CONCLUSION

N_2_O abuse is a noteworthy emerging public health problem that primarily affects young people. Clinicians should be fully aware of the various clinical symptoms that may develop in cases of N_2_O abuse, particularly in young patients with limb numbness and/or weakness, and history of N_2_O exposure should be ascertained in such patients. N_2_O withdrawal and vitamin B_12_ supplementation result in good outcomes, especially for female patients.

## CONFLICT OF INTEREST

The authors declare that they have no known competing financial interests or personal relationships that could have appeared to influence the work reported in this paper.

## FUNDING

This research was funded by the Clinical Research Project of Shengjing Hospital (Reference: M0463) and by the Shenyang Science and Technology Program (Reference: 20‐205‐4‐090).

## AUTHOR CONTRIBUTION

M. Y. and Z. M. contributed to conception and design of the study. F. Y. organized the database. Y. M. performed the statistical analysis. Y. M. and F. Y. wrote the first draft of the manuscript. L. S., G. H., Q. Y., and Z. M. wrote sections of the manuscript. All authors contributed to manuscript revision, read, and approved the submitted version.

### PEER REVIEW

The peer review history for this article is available at https://publons.com/publon/10.1002/brb3.2533

## Data Availability

The data that support the findings of this study are available from the corresponding author upon reasonable request.
